# Phylogeography of *Morella nana*: The Wumeng Mountains as a natural geographical isolation boundary on the Yunnan‐Guizhou Plateau

**DOI:** 10.1002/ece3.11566

**Published:** 2024-07-09

**Authors:** Min Wu, Yu Cheng, Chunxue Jiang, Mingsheng Zhang, Tian Shi, Cai Zhao

**Affiliations:** ^1^ Key Laboratory of Plant Resource Conservation and Germplasm Innovation in Mountainous Region (Ministry of Education), Collaborative Innovation Center for Mountain Ecology and Agro‐Bioengineering (CICMEAB), College of Life Sciences/Institute of Agro‐Bioengineering Guizhou University Guiyang China

**Keywords:** genetic differentiation, *Morella nana*, phylogeography, Wumeng Mountains, Yunnan‐Guizhou Plateau

## Abstract

The Yunnan‐Guizhou Plateau (YGP) is characterized by the distinctive isolated habitat of the limestone Karst Islands and features the Wumeng Mountains, which divide the YGP into the two Plateaus of Yunnan and Guizhou. This study aimed to assess the effects of geographic isolation and past climate fluctuation on the distribution of flora in the YGP. To achieve this, we carried out the phylogeographical pattern and genetic structure based on chloroplast and nuclear ribosomal DNA sequence in relation to past (Last Glacial Maximum) and present distributions based on ecological niche modeling for *Morella nana*, an important wild plant resource and endemic to the YGP once considered a vulnerable species. The results suggest that the genetic and chlorotype network structures of *M. nana* are divided into at least two groups: cpDNA chlorotype H2 (or dominant nrDNA haplotypes h1 and h2), distributed primarily to the east of the Wumeng Mountains, and cpDNA chlorotypes H1 and H3–H10 (or dominant nrDNA haplotype h2 and h3), distributed to the west of the Wumeng Mountains. A deep genetic split was noted within the two groups to reach 25 steps, especially for the cpDNA fragment variation. This east–west divergence reveals the existence of a natural geographical isolation boundary in the form of the Wumeng Mountains, and supports the existence of at least two glacial refuges during the Quaternary glacial period, along with two genetic diversity center, and at least two large geographic protection units for the important species of *M. nana.* This study indicates that the phylogeographical pattern of *M. nana* can be attributed to geographic/environmental isolation caused by the Wumeng Mountains and climate fluctuation during the last glacial maximum, and proposes an effective strategy to protecting this important plant resource.

## Graphical abstract

The east–west divergence reveals the existence of a natural geographical isolation boundary in the form of the Wumeng Mts, which divides the YGP into the Yunnan and Guizhou Plateaus. There existed at least two glacial refugia during the Quaternary glacial period, along with a genetic diversity center, and at least two large geographic protection units for the important species of *M. nana*, distributed throughout the eastern and western sides of the Wumeng Mts. This study not only clarifies that the phylogeographical pattern and for *M. nana* can be attributed to geographic and environmental isolation and climate fluctuation, but it also proposes an effective strategy to protect important wild plant resources.

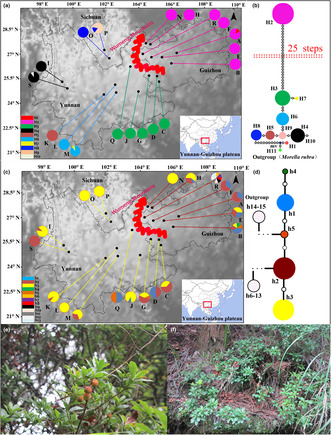

## 1 INTRODUCTION

The evolutionary history and genetic structure of extant species have been greatly affected by both geographic isolation due to the uplift of mountain chains and climatic fluctuations associated with glacial oscillations (Hewitt, 2000). Thus, the distribution patterns and population genetic structures of some species have been reshaped due to geographic barriers and changes in climate (Shahzad et al., 2017). In addition, remarkable climatic fluctuations during the Quaternary ice age were responsible for the range contraction/expansion and isolation of populations in different areas, which resulted in lower genetic diversity and population divergence via bottlenecking and genetic drift (Hewitt, 1996, 2000; Petit et al., 2003; Tian et al., 2015). Genetic diversity is the basis of biological diversity (Linde & Selmes, 2012; Ndjiondjop et al., 2017; Rodríguez‐Nevado et al., 2018), since the extent of genetic diversity directly influences the survival rate of a species, and consequently, its evolution. For example, when the genetic diversity of a species decreases, its survival ability, adaptability, immunity, and coping mechanism with respect to external factors also decrease (Ji & Su, 1999). Studying the genetic diversity of plant species is important for understanding the mechanisms of plant population evolution, adaptation, species origin, and diversity conservation, and can provide effective theoretical guidance for the development of reasonable diversity conservation measures (Livia et al., 2019).

Southwest China is one of the most important biodiversity hotspots and is characterized by extremely complex and geographically isolated habitats (He & Jiang, 2014; Ju et al., 2017). Over the past few years, an increasing body of molecular phylogeographic studies has begun to elucidate how plant species occurring in Southwest China responded to geographic isolation and climatic oscillations during the Quaternary ice age, and have revealed many interesting patterns of genetic diversity and population structure (Liu et al., 2012; Qiu et al., 2011). Several important phylogeographic lines (e.g., the “Tanaka‐Kaiyong Line” and “Mekong‐Salween Divide”) were suggested to have existed in Southwest China, which served as barriers for species to disperse and shaped the phylogeographic patterns of species whose range extended across these lines (Fan et al., 2013; Zhang et al., 2006). Did other phylogeographic lines exist in this range? If so, how did they mold the phylogeographic patterns, genetic structure, and distribution of species in the range?

The Yunnan‐Guizhou Plateau (YGP) in Southwest China is one of four major plateaus in the country. The Wumeng Mountains (Mts), distributed from N25°40′ to 27°30′, E103°32′ to 105°40′, are the obvious cause for geographic isolation of the YGP. Though many regard the Wumeng Mts to be the natural geographical isolation boundary between the Yunnan and Guizhou plateaus, others disagree because there is no accurately identifiable geological and geomorphic boundary between them (Chen & Wang, 2017), necessitating further studies and scientific evidence. Moreover, the YGP is one of the most typical limestone Karst landforms in the world and features a special isolated habitat of Karst Islands (Xu, 2019; Zhao & Chen, 1999). How did this special isolated habitat of Karst Islands shape the genetic structure of species in this region? The obvious spatial and ecological isolation of the YGP makes it an ideal region to study genetic variations and phylogeographical patterns in plants, and researchers have previously identified an east–west divergence in the phylogeographical pattern for some species, such as *Juglans regia* subsp. *sigillata*, *Urophysa rockii*, and *Amorphophallus yunnanensis* (Gao et al., 2024; Sun et al., 2019; Xie et al., 2017). Despite this, studies on the YGP remain sparse, and little is known about the effects of the geographic isolation caused by the Wumeng Mts on the phylogeographic patterns and genetic structure of plants in this area. Thus, a comprehensive and systematic assessment of the impact of geographical and environmental isolation on the phylogenetic relationship, differentiation and formation, and geographical distribution patterns of the plant species in this region is imperative.

Molecular approaches are effective in studying the genetic structure and evolution of extant species and are independent from fossil information (Avise, 2000; Tian et al., 2009). Chloroplast DNA (cpDNA) is particularly useful due to its small molecular weight, multiple copies, simple structure, independent evolution, and relatively quick evolution of non‐coding regions and has been used in phylogeographical studies to understand the impact of geographic variation on plant evolution (Olmstead & Palmer, 1994; Sun et al., 2019; Wicke et al., 2011). Nuclear ribosome DNA (nrDNA) contains highly repetitive tandem sequences and consists of three external transcribed spacers (ETS): 18S, 5.8S, and 26S, and two internal transcribed spacers (ITS): 18S and 5.8S. The intergenic regions between 18S and 5.8S, and 5.8S and 26S are called the first transcription spacer (ITS1) and the second transcription spacer (ITS2), respectively (Álvarez & Wendel, 2003; Li et al., 2011; Schoch et al., 2012; Zhang et al., 2022). These ITS fragments of nrDNA can provide a large amount of information about species, due to the advantages of small length variation, fast base replacement rate, and easy amplification and sequencing, and are widely used in phylogeographic research (Zhang et al., 2022).

In this study, we sequenced two cpDNA sequences (*psbA*–*trnH* and *trnD*–*psbM*) and nrDNA (ITS) sequences and used ecological niche modeling to investigate the phylogeographic pattern and genetic variation in *Morella nana* (A. Chev.) J. Herb., distributed in the YGP, with 19 populations occurring across the Wumeng Mts. *M. nana*, also known as Yunnan bayberry, is a wild evergreen shrub belonging to the Myricaceae family. It is an important wild plant resource and was once considered a vulnerable species (Liu & Li, 1998; Liu & Li, 2003; Xiao et al., 1997). It is endemic to the YGP, found in northeastern and central Yunnan, ranging from the southwest of Guizhou to the southern edge of Sichuan and spanning the east and west of the Wumeng Mts (Liu & Li, 2003; Wu, 1972). *M. nana* has high medicinal and economic value, and can potentially be used for three‐dimensional greening and production, or as the pioneer tree species for restoring the mountain vegetative cover that was depleted due to coal mining (Liu & Li, 2003; Xiang, 2005). The Myricaceae is a small, sub‐cosmopolitan family of about 50 species, but only one genus and four species are found in China: *M*. *rubra* Lour., *M*. *nana*, *M*. *adenophora* (Hance) J. Herb, and *M*. *esculenta* (Buch.‐Ham. ex D. Don) I. M. Turner. Phylogenetic studies have strongly supported the monophyly of these four species, and the relationships among them have been resolved (Liu et al., 2016). The genetic diversity and population structure of wild *M*. *rubra* populations have been revealed based on genomic data (*H*_d_ = 0.1950; Liu et al., 2016). However, little is known about the evolutionary history and genetic structure of *M. nana* in the YGP (Liu, Chen, & Li, 2003; Liu & Li, 1998; Liu, Li, & Yi, 2003). In this study, our specific aims were to: (1) detect the consequences of the phylogeographical pattern and demographic history of *M. nana*, and investigate the causes leading to the consequences—geographical isolation (Wumeng Mts and/or the isolated habitats of the unique Karst Islands) and/or the advent of the Quaternary glacial period and the subsequent periodic fluctuations, among other factors, (2) address the scientific debate on whether the Wumeng Mts are the natural geographical isolation boundary between the Yunnan and Guizhou plateaus on the YGP, based on the phylogeographical pattern of *M. nana*, (3) resolve whether the populations within the region came from a common shelter or from separate shelters within each region during the Quaternary ice age, and (4) outline strategies for protecting *M. nana*, an important wild plant resource that was once considered a vulnerable species in the YGP.

**FIGURE 1:**
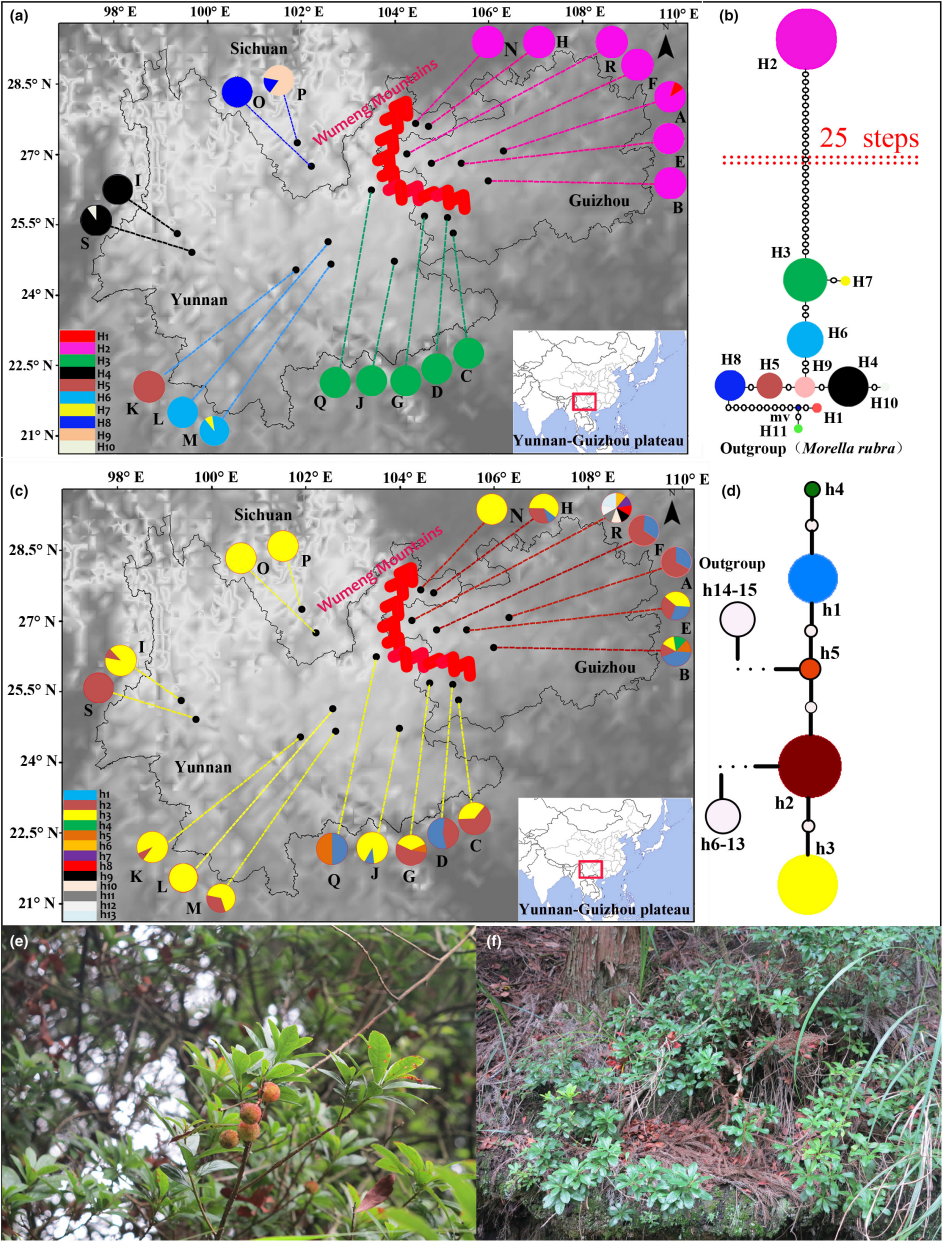
Wild *Morella nana* and map of sampling locations in Table 1, the location of Wumeng Mountains, and the geographic distribution and network of chlorotypes detected in different populations. (a) Sampling locations in Table 1 and the geographic distribution of cpDNA chlorotypes and the location of Wumeng Mountains. All haplotypes found in each population and isolated groups are color‐coded and displayed; (b) Network of cpDNA chlorotypes and groups. (c) Sampling locations in Table 2 and the geographic distribution of nrDNA haplotypes and the location of Wumeng Mts. All haplotypes found in each population and isolated groups are color‐coded and displayed. (d) Network of nrDNA haplotypes and groups. Circle sizes represent the frequency of each haplotype. Hollow dots display inferred intermediate chlorotypes that are not detected in our research or are extinct (b, d). Each solid line indicates one mutational step interconnecting two chlorotypes. *M. rubra* is included as the outgroup; (e, f) present the branches with fruit plant, respectively.

## 2 MATERIALS AND METHODS

### 2.1 Sample collection

A total of 197 samples of *Morella nana* were collected from 19 natural and wild *M. nana* populations encompassing the entire geographical distribution range of *M. nana* in the YGP and occurring across the Wumeng Mts (Table 1, Figures 1 and 3). Each population included 2–12 individual samples that were collected at least 50 m apart. The isolated distance among populations was at least 20 km. The collected leaves were cleaned, gathered into a molecular material collection bag, numbered, and placed inside a storage device containing silica gel for drying and preservation. The collected branches for each sampled population were labeled with relevant information (latitude, longitude, altitude) and processed to obtain the specimens. Voucher specimens were numbered from ZhaocA1~3 to ZhaocS1~3 and deposited in the plant herbarium at the College of Life Science at Guizhou University. According to a phylogenetic study of Chinese *Morella* by Liu et al., *M. rubra* is closely related to *M. nana* (Liu et al., 2016). Thus *M. rubra* is included as an outgroup in our study.

**TABLE 1:** Information of sample location and sample size of *Morella nana*.

Population code	Sampling location	Longitude (E)	Latitude (N)	Altitude (m)	Sample	CpDNA chlorotypes (no. of individuals)									
						H1	H2	H3	H4	H5	H6	H7	H8	H9	H10
A	Bijie, Guizhou	106°03′	27°15′	1410	9	1	8								
B	Bijie, Guizhou	105°44′	26°37′	1490	7		7								
C	Xingren, Guizhou	105°58′	25°39′	1860	11			11							
D	Liuzhi, Guizhou	105°14′	26°23′	1590	8			8							
E	Shuicheng, Guizhou	104°59	26°30′	1720	10		10								
F	Yangmei, Guizhou	104°48′	26°23′	2250	2		2								
G	Panzhou, Guizhou	104°29′	25°39′	2150	9			9							
H	Zhaotong, Yunnan	104°16′	27°72′	1662	12		12								
I	Dali, Yunnan	100°23′	25°58′	1992	11				11						
J	Wenshan, Yunnan	103°86′	24°04′	2090	10			10							
K	Yuxi, Yunnan	102°40′	24°18′	1542	12					12					
L	Kunming, Yunnan	102°58′	25°63′	1753	5						5				
M	Kunming, Yunnan	102°80′	25°74′	2015	12						11	1			
N	Zhaotong, Yunnan	104°13′	27°38′	1851	12		12								
O	Panzhihua, Sichuan	101°86′	26°69′	1143	11								11		
P	Panzhihua, Sichuan	101°74′	26°51′	1099	11								2	9	
Q	Qujing, Yunnan	103°69′	26°63′	2467	3			3							
R	Bijie, Guizhou	104°14′	26°88′	2209	9		9								
S	Dali, Yunnan	100°45′	25°07′	1401	10				9						1
Total					174	1	60	41	20	12	16	1	13	9	1

*Note*: For each population, frequencies of chlorotypes were estimated based on two cpDNA (*psb*A–*trn*H + *trn*D–*psb*M).

It is worth emphasizing that although the sampling size (each population included 2–12 samples for *M. nana*) was unbalanced between populations in our phylogeographic investigation, this cannot affect the results presented. Sample sizes for *Tetrastigma hemsleyanum* (individuals from 6 to 15), *Machilus thunbergii* (individuals from 2 to 8), *Bombax ceiba* (individuals from 5 to 34) and *Bupleurum smithii* (individuals from 3 to 13, our studies have been published.) were unbalanced in other phylogeographic studies, but it can cover the representative populations and geographical distributions (Fan et al., 2022; Ju et al., 2017; Wang et al., 2015; Zhao et al., 2013). In addition, given that *M. nana* was once a vulnerable species endemic to the YGP, its populations and individual plants within a population are relatively few. Nevertheless, the samples we collected covered the entire geographical distribution of the species and we attempted to collect as many populations and individual plants as possible in our study.

### 2.2 DNA extraction, PCR amplification, and sequencing

Two total DNA extraction methods were employed in the experiment. One total DNA extraction method was adopted from the modified traditional CTAB method, and the Beijing Tiangen plant genomic DNA extraction kit method (Doyle & Doyle, 1987) was also used. When one method ceased to be effective for numerous rounds of extraction, the other method was utilized to increase the total DNA extraction of *M. nana*. The total DNA samples of *M. nana* were detected by agarose gel (1%) electrophoresis. Subsequently, the gel was removed and placed in a gel imager for observation. In the preliminary experiment, three primer pairs for ITS, *psbA*–*trnH*, and *trnD*–*psbM* amplification were selected based on the published sequences and related reports on *M. rubrum* (Liu, 2015; Liu et al., 2016). These primer pairs were amplified stably and had high polymorphic nrDNA fragmentation and cpDNA gene intervals. The primers were synthesized by Qingkexing Biotechnology Co., Ltd. (Chongqing, China). Polymerase chain reaction (PCR) was performed for a total volume of 25 μL. The two cpDNA regions and nrDNA were amplified, and the amplified products were sequenced by Shuo Qing Biotechnology Co., Ltd. (Kunming, China). The PCR products of *M. nana psbA*–*trnH*, *trnD*–*psbM*, and ITS fragments were detected via 1% agarose gel electrophoresis, which was similar to the total DNA detection technique utilized for *M. nana*. The PCR primers and amplification procedures are shown in Table S1.

### 2.3 Data analysis

#### 2.3.1 Sequencing result processing

Chromas (Technelysium Pty Ltd, https://technelysium.com.au/wp/chromas) was employed to visualize the sequence peak diagram of the sequenced DNA fragments, and SeqMan 11.2 software in DNAstar (Burland, 1998) was utilized for sequence editing and assembly. MEGA v 7.0 software (Sudhir et al., 2016) was used to perform multiple sequence alignment analysis, correct the wrong bases, and perform para‐position sequencing on the sequences. Moreover, the sequence primer areas were manually corrected and removed from both ends. The corrected DNA fragments were stored in a uniform format.

#### 2.3.2 Analysis of population genetic diversity and haplotype phylogeny

Haplotypes were identified and differentiated using the DnaSP 5.0 software package (Librado & Rozas, 2009). Haplotype and nucleotide diversities were calculated for each population and at the species level (*H*_d_, *π*_d_) using DnaSP v 5.0. To quantify the total genetic variation and the level of variations between different groups, analysis of molecular variance (AMOVA) in the ARLEQUIN software package (Excoffier et al., 2005) was used to analyze the cpDNA nrDNA datasets. This approach considers both differences in sequence frequencies and the number of variable sites among the observed sequences. A 1000 random permutation and analysis test was applied to discern the degree of differentiation between populations. The median‐joining network method (Bandelt et al., 1995, 1999) run on NETWORK v 4.5 (Polzin & Daneshmand, 2003; available at http://www.fluxus‐engineering.com) and based on the principle of simplicity was used to analyze and construct haplotype network taking the central joining network method for cpDNA and nrDNA haplotypes. After generating the initial median‐joining network, excessive links and median vectors were chosen to add. At the same time, to obtain accurate and reliable results, we used software TCS v1.21 (Clement et al., 2010) to supplement and verify the constructed haplotype intermediate network. Neighbor‐joining (NJ) analyses with 1000 replicates and Kimura 2‐parameter model were conducted using MEGA v 7.0. (Sudhir et al., 2016). We also used maximum likelihood (ML) analyses in MEGA v 7.0 to construct the relationships of all chlorotypes. We carried out ML heuristic search parameters through the simple addition of sequences of taxa with Tree‐Bisection Reconnection (TBR) branch swapping, and heuristic searches were carried out with 1000 random addition sequence replicates. Bootstrap values were estimated to appraise the relative support for relationships between chlorotypes (1000 replicates). Maximum Parsimony (MP) analyses were carried out using MEGA v 7.0. All characters were considered unordered and equally weighted. Gaps were considered to be missing data. Heuristic searches were carried out with 1000 random addition sequence replicates. To estimate clade support, bootstrap values were calculated from 1000 replicate analyses using faster stepwise‐addition of taxa and only those whose values were compatible with the majority‐rule consensus tree were logged.

#### 2.3.3 Dynamic analysis of population history

The historical demography of *M. nana* was characterized using the mismatch distribution analysis (Rogers & Harpending, 1992) implemented in DnaSP v5.0 software (Librado & Rozas, 2009) for each population group and all populations. This was done to detect whether the population groups underwent any recent population amplifications. The distribution of ambiguities indicated that the population did not experience recent expansion, whereas a unimodal curve indicates that the population underwent a recent range expansion. In addition, we also calculated Tajima's *D* (Tajima, 1989) and Fu & Li's *D* (Fu, 1997) to assess possible expansions. The values of two neutral test methods for infinite mutation site models were utilized along with the mismatch analysis to determine whether the population had undergone recent expansion.

Additionally, the Bayesian Skyline Plot method (BSP) was implemented in BEAST v 2.7.6 (Bouckaert et al., 2019) to analyze the population size dynamics through time. We use the average base mutation rates(2 × 10−9 substitutions/site/year) that Wolfe et al. (1987) previously estimated for the angiosperm chloroplast DNA (1~3 × 10^−9^ substitutions/site/year), alongside a population coalescent Bayesian Skyride model for the prior tree and strict molecular clock. A random initial tree, the linear model, and set the lengths of the MCMC chains were used to achieve ESS ≥200 (10,000,000 chains for both cpDNA haplotype datasets). We ran three independent analyses and combined them with TRACER 1.5 to assess related parameters. Finally, the BSP was analyzed and plotted based on the R language. ITS fragments of nrDNA can provide a large amount of information about species, but it is not suitable to carry out analysis of the population size dynamics and divergence through time due to the fast base replacement rate (Shahzad et al., 2017). Thus the datasets were not adopted to conduct the analysis of Bayesian Skyline Plot in our study.

#### 2.3.4 Present and past ecological niche modeling

Ecological niche modeling (ENM) was carried out in MaxEnt 3.4.1 (Feng et al., 2018; Phillips & Dudík, 2008) to predict suitable past (last glacial maximum; LGM) and present climate envelopes for *M. nana*. As a means to estimate *M. nana* species distribution models (SDMs) along Quaternary climatic fluctuations, we implemented the maximum entropy machine‐learning algorithm MAXENT (Fava et al., 2020; Phillips & Dudík, 2008). MAXENT estimates an index of relative habitat suitability that is based on presence‐only data (specimen collection records). The distribution records for *M. nana* were mainly downloaded from the Global Biodiversity Information Facility (GBIF) (https://www.gbif.org/). Combined with the species distribution records (19 points) recorded in this study and the records (118 points) obtained from the literature and GBIF, distribution points less than 10 km apart were removed from ArcGIS 10.2 (Esri, 2013), a total of 137 specimen collection records were looked up and adopted to conduct ecological niche modeling in our study. Then, 19 bioclimatic factors from the current, mid‐Holocene (about 6 kya), Last Holocene (about 22 kya), and future (2050) were downloaded from WorldClim. The variables were derive from monthly temperature and rainfall values, which allowed generation of more biologically meaningful variables (Fava et al., 2020). The bioclimatic variables represent annual trends (e.g., mean annual temperature, annual precipitation), seasonality (e.g., annual range in temperature and precipitation), and extreme or limiting environmental factors (e.g., temperatures of the coldest and warmest month; Fava et al., 2020). To avoid the influence of multicollinearity, Pearson correlation coefficients among bioclimatic factors were calculated in SPSS 2.0, and bioclimatic factors with Pearson correlation coefficients significantly greater than 0.8 were removed. Our study retains one of the relevant variables. When the correlation coefficient of the two environmental factors is greater than 0.8, the environmental factor with less biological significance is selected to be discarded according to the study material. MaxEnt 3.4.1 was used to predict the species distribution range of *M. nana* in different periods. Seven environmental factors were retained: bio3 (Isothermally), bio4 (Temperature Seasonality), bio7 (Temperature Annual Range), bio14 (Precipitation of Driest Month), bio15 (Coefficient of Precipitation variation), bio18 (Precipitation of Warmest Quarter), and bio19 (Precipitation of Coldest Quarter).

## 3 RESULTS

### 3.1 Genetic diversity

The study involved amplification sequencing and splicing of *psbA*–*trnH* and *trnD*–*psbM* in the chloroplasts of 174 individual plants from 19 populations of *M. nana*, and the splicing length of the two segments was 1026 bp. A total of 15 mutation sites were detected by DnaSP analysis. Among them were 12 sites of nucleotide replacement and three sites of insertion/deletion, and a total of 10 chlorotypes, H1–H10, were produced (Tables 1 and S2). There were 15 populations with only one haplotype, and the remaining four populations (chlorotypes H1 and H8 distributed in population A; chlorotypes H6 and H7 distributed in population M; chlorotypes H8 and H9 distributed in population P; chlorotypes H4 and H10 distributed in population S) had two chlorotypes (Table 1 and Figure 1a). The analysis with DnaSP software showed that the overall haplotype diversity (*H*_d_) was 0.7954, while the overall nucleotide diversity (*π*_d_) was 0.00255.

Following amplification, sequencing, correction, and splicing of 163 individual samples from the 19 populations of *M. nana*, the ITS gene fragment length was determined to be 553 bp. A total of 23 mutation sites were analyzed and detected through the DnaSP software, and all these sites presented nucleotide substitution without site deletion, resulting in a total of 13 haplotypes, h1–h13 (Tables 2 and S3). Population R contained the greatest number of haplotypes (8 haplotypes). Most other populations generally contained one or two haplotypes, while populations B, E, G, and H contained three to five haplotypes. The overall haplotype diversity (*H*_d_) was 0.6690, and the overall nucleotide diversity (*P*i) was 0.00477.

**TABLE 2:** Information of sample location and sample size of *Morella nana*.

Population code	Sampling location	Longitude (E)	Latitude (N)	Altitude (m)	Sample	nrDNA ITS haplotypes (no. of individuals)												
						h1	h2	h3	h4	h5	h6	h7	h8	h9	h10	h11	h12	h13
A	Bijie, Guizhou	106°03′	27°15′	1410	9	3	6											
B	Bijie, Guizhou	105°44′	26°37′	1490	7	3	1	1	1	1								
C	Xingren, Guizhou	105°58′	25°39′	1860	11		7	4										
D	Liuzhi, Guizhou	105°14′	26°23′	1590	9	5	4											
E	Shuicheng, Guizhou	104°59	26°30′	1720	10	3	3	4										
F	Yangmei, Guizhou	104°48′	26°23′	2250	8	3	5											
G	Panzhou, Guizhou	104°29′	25°39′	2150	11		6	4		1								
H	Zhaotong, Yunnan	104°16′	27°72′	1662	10	1	3	6										
I	Dali, Yunnan	100°23′	25°58′	1992	10		1	9										
J	Wenshan, Yunnan	103°86′	24°04′	2090	10	1		9										
K	Yuxi, Yunnan	102°40′	24°18′	1542	12		1	11										
L	Kunming, Yunnan	102°58′	25°63′	1753	5			5										
M	Kunming, Yunnan	102°80′	25°74′	2015	12		4	8										
N	Zhaotong, Yunnan	104°13′	27°38′	1851	12		10	2										
O	Panzhihua, Sichuan	101°86′	26°69′	1143	4			4										
P	Panzhihua, Sichuan	101°74′	26°51′	1099	2			2										
Q	Qujing, Yunnan	103°69′	26°63′	2467	2	1				1								
R	Bijie, Guizhou	104°14′	26°88′	2209	9						1	1	1	1	1	1	1	2
S	Dali, Yunnan	100°45′	25°07′	1401	10		10											
Total					163	20	61	69	1	3	1	1	1	1	1	1	1	2

*Note*: For each population, frequencies of haplotypes were estimated based on nrDNA ITS fragments.

### 3.2 Haplotype interrelationship

Based on the two cpDNA fragments (*psbA‐trnH* and *trnD‐psbM*), TCS v 1.21 software and NETWORK v 4.5 software were used to obtain a chlorotype geographical distribution and haplotype network map (Figure 1a,b). Ten chlorotypes were identified for all sampled individuals of *M. nana*, following which NJ/ML/MP phylogenetic trees were constructed for the 10 chlorotypes (Figures S1–S3, S7). H2 and H3 were two chlorotypes with the widest geographical distributions and highest frequencies. The occurrence frequency of H2 was 36.8% in all populations and was distributed east of the Wumeng Mts. Meanwhile, the occurrence frequency of H3 was 26.3%; less than H2, and distributed to the southwest and west of the Wumeng Mts. The remaining chlorotypes were observed to the west of the Wumeng Mts. The steps between H2 and H3 were found to be up to 25, indicating a long time since the divergence of these two chlorotypes. There were numerous mutations identified between the two chlorotypes, which might be extinct at present, and more populations with only one native chlorotype, which might indicate that *M. nana* experienced a population retreat and genetic bottleneck in its long evolutionary history. Chlorotype H2 could be divided into a cluster (Group I) by combining the geographical distribution and network of chlorotypes. Chlorotypes H6 and H7 were divided into first‐order branches based on H3. Other chlorotypes, however, were divided from the H6 haplotype into second‐order branches and multistage branches, with small steps. Therefore, combined with the distribution and network of chlorotypes, chlorotypes H1, H3 to H10 could be regarded as a group (Group II). It is worth mentioning that in the geographic distribution map, chlorotype H1 was presented on the eastern side of the Wumeng Mts, while in the haplotype network map, chlorotype H1 was derived from haplotype H4, and was distributed among the western group on the YGP. The chlorotypes H2, H3, H6, and H7 were all in one clade and occupied the main position in the center and to the east of the YGP; the distribution frequencies of chlorotypes H2, H3, and H6 were relatively high. Chlorotypes H4, H9, and H10 were uniformly distributed as a single clade located at the edge of the YGP in the geographical distribution map. Meanwhile, the chlorotypes H1 and H8 were native chlorotypes and were distributed in populations A and S, respectively (Table 1 and Figure 1a).

Haplotype geographical distribution and haplotype network structure were analyzed based on the nrDNA fragments (ITS), and an NJ/ML/MP phylogenetic tree was constructed for 13 haplotypes (Figures S4–S6, S8). Haplotypes h1, h2, and h3 exhibited the widest distribution and exceedingly high frequencies of occurrence – up to 12.27%, 37.42%, and 42.33%, respectively. Haplotypes h2 and h3 were mainly distributed to the west of the Wumeng Mts, while haplotypes h1, h2, and the other native haplotypes mainly occurred to the east of the Wumeng Mts on the YGP (Table 2 and Figure 1c). The haplotype interrelationship in the network structure was consistent with the result of the haplotype phylogenetic tree.

### 3.3 Population historical dynamics

**FIGURE 2:**
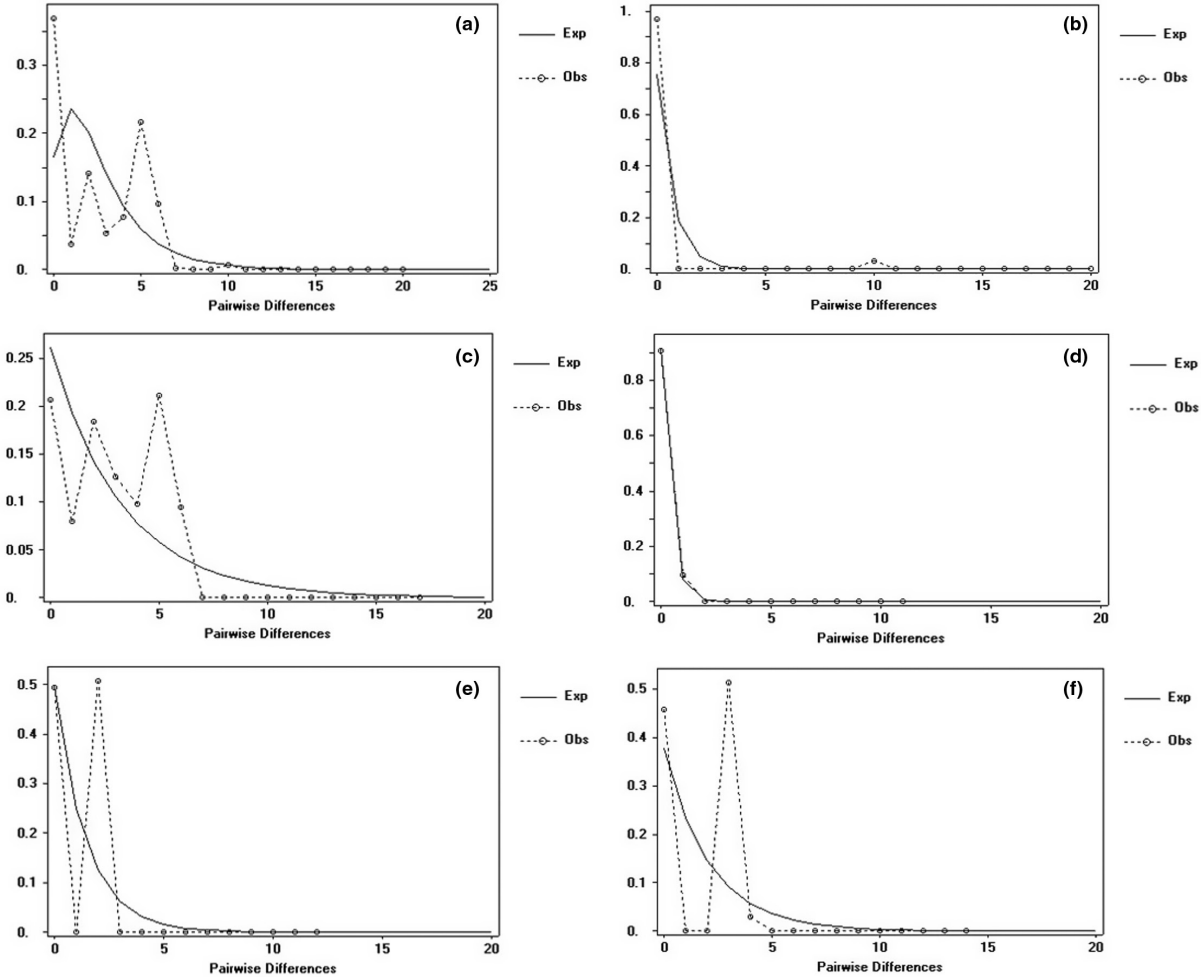
Mismatch distribution of cpDNA (*psb*A–*trn*H *+ trn*D–*psb*M) sequences of *Morella nana*. The line represents distributions expected for expansion, and the dotted line represents the observed mismatch distribution. (a) Yunnan‐Guizhou Plateau region; (b) Guizhou Plateau region (east of the Wumeng Mountains); (c) Yunnan Plateau region (west of the Wumeng Mountains); (d) Dali region; (e) Panzhihua region; (f) Central region.

The neutral detection and mismatch analysis of the chloroplast genes of *M. nana* was carried out using DnaSP v5.0 software. Mismatch analysis is a means to reflect the historical dynamics of a population. If the population has undergone expansion, the mismatch distribution curve would exhibit a single peak, underlining the significance of the neutral test. If the population size has remained stable, the mismatch distribution curve presents a multi‐peak feature, and the neutral test is not significant. This study included the analysis of overall and regional populations based on the results of the geographical distribution and the network of chlorotypes across the entire YGP region (a: populations A–S), the Guizhou Plateau region (b: populations A, B, E, D, H N, and R), the Yunnan Plateau region (c: populations C, D, G, I, J, K, L, M, O, P, Q, and S), the Dali region (d: populations I and S), the Panzhihua region (e: populations O and P), and the central region (f: populations K, L, and M). In terms of mismatch analysis, it is evident from a, c, e, and f that the actual curve exhibited multiple peaks (Figure 2a,c,e,f), which is significantly different from the expected curve. The results showed that the *M. nana* populations experienced no obvious expansion in these four regions. In contrast, we can see the observed and expected value curves in b and d both showed an unimodal distribution with a downward trend (Figures 2b,d), indicating that the populations in these two areas have recently undergone region expansion. In addition, it is evident from the neutral test of population division in each region that the neutral test value of *D* in d (Dali region) was negative (Table 4). This indicates population expansion in those regions, which is consistent with the results of the mismatch analysis. However, there were three significant negative values in the neutral monitoring in b (Guizhou Plateau region; Table 3). Although Fu's *F* value was positive, it did not fully explain the hypothesis that this region deviates from expansion. Neutral Tajima's *D* and Fu's *F* were positive in a and f. However, the values of Fu and Li's D* and Fu and Li's F* were both negative and the values were not significant (*p* > .10), thus indicating that the region has not experienced significant expansion. The BSP indicated that the species experienced region population size expansion after 0.072 Ma based on cpDNA datasets (Figure S9).

**FIGURE 3:**
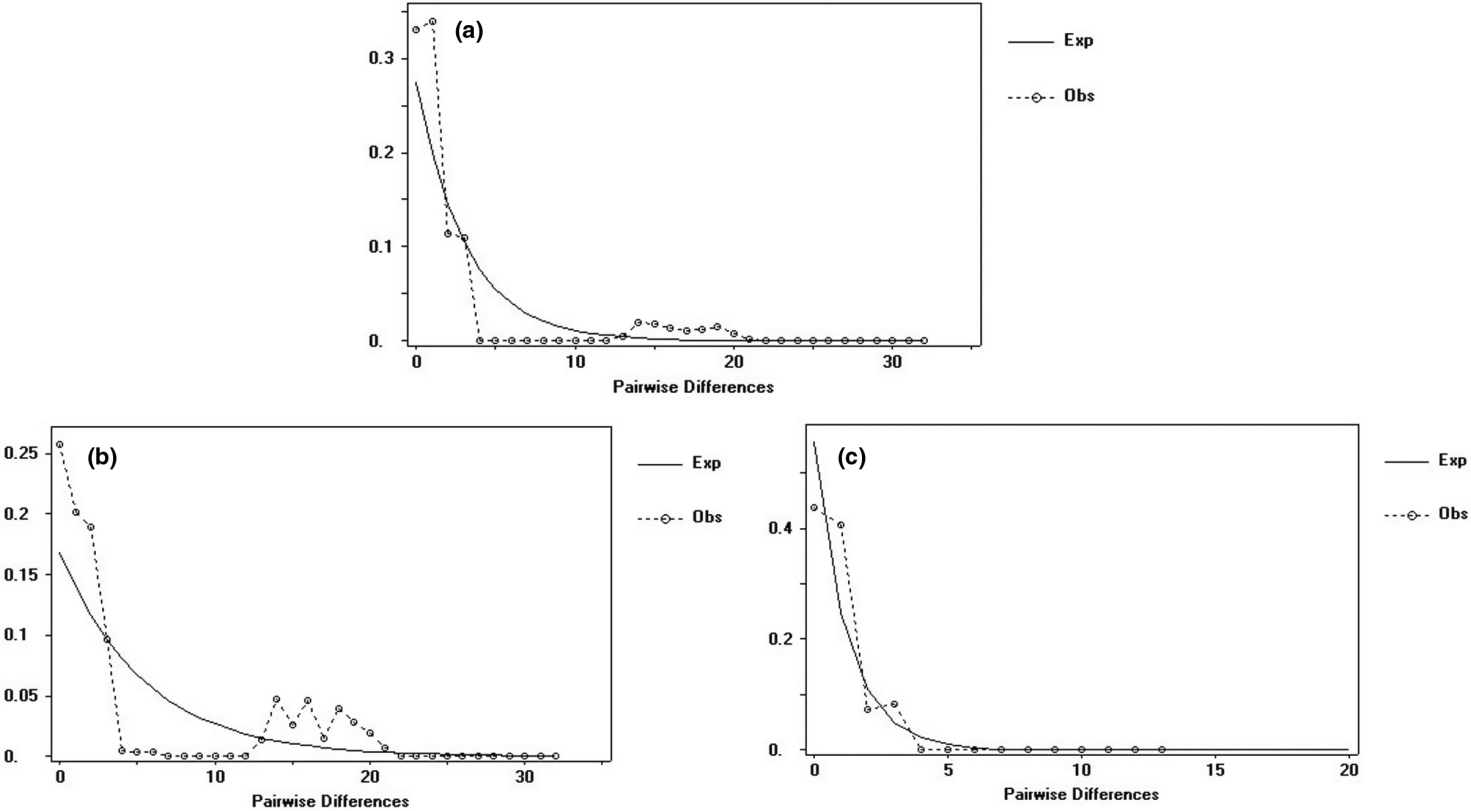
Mismatch distribution of ITS intergenic spacer of *Morella nana*. The line represents distributions expected for expansion and the dotted line represents the observed mismatch distribution. (a) all populations; (b) populations distributed east of the Wumeng Mountains; (c) populations distributed west of the Wumeng Mountains.

**TABLE 3:** The neutral test of *Morella nana* in different area based on cpDNA (*psbA–trnH* + *trnD–psbM*) sequences.

Area	Neutral test			
	Tajima's *D*	Fu and Li's D*	Fu and Li's F*	Fu's *Fs*
Yunnan‐Guizhou Plateau region (a)	0.5255 (*p* > .10)	−1.98359 (.10 > *p* > .05)	−1.26734 (*p* > .10)	1.622
Guizhou Plateau region (b)	−2.33784 (*p* < .01)	−5.01857 (*p* < .02)	−4.86664 (*p* < .02)	1.194
Yunnan Plateau region (c)	2.09453 (*p* < .05)	0.42943 (*p* > .10)	1.18927 (*p* > .10)	2.251
Dali region (d)	−1.16356 (*p* > .10)	−1.55770 (*p* > .10)	−1.66344 (*p* > .10)	−0.919
Panzhihua region (e)	1.91752 (.10 > *p* > .05)	0.85062 (*p* > .10)	1.31254 (*p* > .10)	3.081
Central region (f)	0.82575 (*p* > .10)	−0.59275 (*p* > .10)	−0.20389 (*p* > .10)	3.135

The neutral test and mismatch analysis were carried out based on the ITS and population of three distribution regions (a, b, and c). Out of the *M. nana* populations A–S on the YGP, the populations A, B, E, F, R, N, and H were distributed to the east of the Wumeng Mts and the populations C, D, G, I, J, K, L, M, O, P, Q, and S were distributed to the west of the Wumeng Mts, as discerned by DnaSP v5.0 software. Multi‐peak characteristics were detected for a, b, and c in the mismatch distribution curve, which is consistent with the result of the neutral test. Moreover, the results indicated that the population of *M. nana* had not experienced expansion in the above regions (Table 4, Figure 3). It can be speculated that the colony of *M. nana* has been preserved in situ throughout its long evolution history and that there was a brief and small expansion within group populations.

**TABLE 4:** The neutral test of *Morella nana* in different areas based on nrDNA sequences.

Areas	Neutral test			
	Tajima's *D*	Fu and Li's D*	Fu and Li's F*	Fu's *Fs*
a (all of populations)	−0.97594 (*p* > .10)	1.83309 (*p* < .02)	0.86420 (*p* > .10)	−0.645
b (populations distributed in the east of Wumeng Mountains)	0.06046 (*p* > .10)	1.77072 (*p* < .02)	1.37153 (*p* > .10)	0.555
c (populations distributed in the west of Wumeng Mountains)	0.67685 (*p* > .10)	0.82939 (*p* > .10)	0.91623 (*p* > .10)	0.789

### 3.4 Population genetic structure

Analysis of molecular variance (AMOVA) was carried out for all individuals in the population groups. On the one hand, at least two groups, Group I (populations A, B, E, F, H, N, and R) and Group II (populations C, D, G, I, J, K, L, M, O, P, Q, and S), were divided according to geographical distribution and the network structure of chlorotypes (Figure 1). These groups were distributed to the east and west of the Wumeng Mts, respectively. On the other hand, all the populations on the YGP were divided into five groups, the Dali area (populations I and S), the central Yunnan Plateau (populations L, M, and K), the Panzhihua area (populations O and P), the eastern Wumeng Mts (populations A, B, E, F, H, N, and R), and the southwestern Wumeng Mts (populations C, D, G, J, and Q) based on the geographical distribution and types of chlorotypes (Figure 1 and Table 1). A higher proportion of genetic variation between groups was found in these two grouping methods, and the genetic variation among the groups was up to 90.58% and 94.16% (Table 5), respectively; higher than the intergroup and within‐population genetic variation. It is evident from the results that the genetic variation of *M. nana* based on ITS was mainly attributed to inter‐population genetic variation. Furthermore, the proportion of genetic variation ratio was 63.62%, while it was 29.14% within the population (Table 6).

**TABLE 5:** The AMOVA analysis based on cpDNA (*psb*A*–trn*H + *trn*D*–psb*M) sequences for *Morella nana*.

Source of variation	Degree of freedom	Sum of squares	Variance components	Percentage of variation
Geographic group 1				
Among groups	1	970.719	12.12466	90.58*
Intergroup	17	155.810	0.98132	7.33
Within‐population	155	43.367	0.27989	2.09
Total	173	1169.897	13.38577	
Geographic group 2				
Among groups	4	1094.808	8.16488	94.16*
Intergroup	14	31.722	0.22670	2.61
Within‐population	155	43.367	0.27979	3.23
Total	173	1169.897	8.67136	

*Note*: Geographic group 1: group I (containing populations C, D, G, I, J, K, L, M, O, P, Q, and S); group II (contain populations A, B, E, F, H, N, and R). Geographic group 2: group I (containing populations I and S); group II (contain populations L, M, and K); group III (contain populations O and P); group IV (containing populations A, B, E, F, H, N, and R); group V (contain populations C, D, G, J, and Q).

* *p* < .05.

**TABLE 6:** The AMOVA analysis based on nrDNA sequences for *Morella nana*.

Source of variation	Degree of freedom.	Sum of squares	Variance components	Percentage of variation
Among groups	1	16.952	0.09991	7.04
Intergroup	17	137.245	0.90625	63.62*
Within‐population	144	59.583	0.41377	29.14*
Total	162	213.779	1.41993	

*Note*: Geographic group: group (containing populations A~S).

* *p* < .05.

### 3.5 Analysis results of ecological niche modeling

**FIGURE 4:**
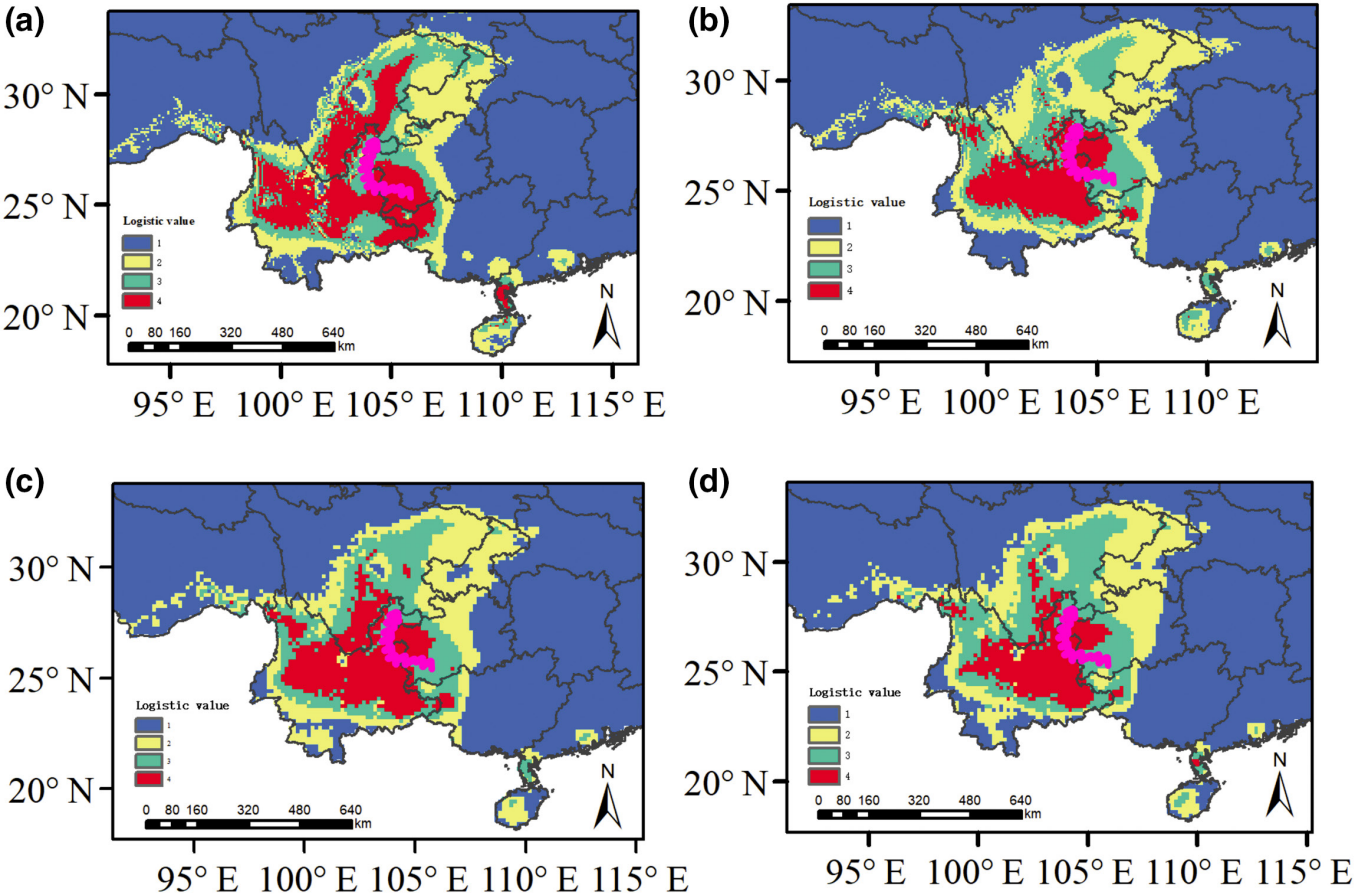
Results of species distribution model in *Morella nana*. (a) Last glacial maximum (LGM); (b) Mid‐Holecene; (c) Current‐day; (d) Future (2050). The pink stripe represents the location of the Wumeng Mountains.

The climatic factors screened by Pearson correlation analysis were used to reconstruct the historical distribution area during the present time, the last glacial maximum (LGM), the middle Holocene and the future for *M. nana* based on ENM. The results showed that during the LGM, the distribution range of suitable areas for *M. nana* was shrunk into two main regions, which is consistent with the Guizhou Plateau and Yunnan Plateau. The distribution range of suitable areas in the Yunnan Plateau further contracted into several small areas. The suitable area expanded slightly from the Middle Holocene to the present distribution range, and then a second contraction between the Guizhou Plateau and Yunnan Plateau occurred. The expansion area was mainly concentrated southwest, and the suitable area showed a small contraction trend in these areas in the future (Figure 4). Among the selected environmental variables, the knife cut test results showed that the environmental variable with the greatest influence on the distribution rate of *M. nana* was the warmest seasonal precipitation (bio18) in the LGM, the middle Holocene, the modern period, and the future four periods, indicating that the warmest seasonal precipitation had a great influence on the distribution of *M. nana*.

## 4 DISCUSSION

### 4.1 Genetic diversity on cpDNA and nrDNA

Genetic diversity is an important component of biological diversity (Ndjiondjop et al., 2017). Genetic diversity in a broad sense refers to the total amount of genetic information carried by organisms on earth, which is stored in the genes of individual organisms. In the narrow sense, genetic diversity refers to the variation of genes within a population, including genetic variation between significantly different populations and within the same population. Research has suggested that plant populations with high levels of genetic diversity have a stronger ability to adapt to ecological and environmental changes and are more likely to expand their distribution patterns, while populations with low levels of genetic diversity are more likely to be affected by internal genetic factors and external environmental changes (Liu et al., 2020). In the present study, 10 chlorotypes of cpDNA were detected based on *psbA‐trnH* + *trnD‐psbM* fragments for *M. nana*. These populations showed medium levels of chlorotype diversity (*H*_d_ = 0.7954) and nucleotide diversity (*P*_i_ = 0.00255), which was greater than that of *M. rubra* (0.1950 and 0.00033; Liu et al., 2016). Thirteen chlorotypes were obtained based on nrDNA fragments for *M. nana*, and the haplotype diversity and nucleotide diversity were 0.6690 and 0.00477, respectively. The cpDNA variance showed moderate levels of chlorotype diversity and lower levels of nucleotide diversity (*P*_i_ = 0.00255) for *M. nana* in the entire geographical region. These results are similar to previous studies on plants in East Asia, and near to the average value of 0.67 determined for 170 plant species (Petit et al., 2010). Notably, the *H*_d_ value for *M. nana* is higher than that of some plants on the YGP Such as, *Juglans regia* subsp. *sigillata* (0.203), *Amorphophallus yunnanensis* (0.341), and *Urophysa rockii* (0.742; Gao et al., 2024; Sun et al., 2019; Xie et al., 2017). Low chloroplast gene diversity in general shows that species have a relatively short evolutionary history and significant effects of the ice age (Ge, 2001; Hewitt, 2000). Both *M. nana* and *M. rubra* belong to Myricaceae, but they exhibited different chloroplast haplotype polymorphisms. The haplotype polymorphism value of *M. rubra* was very low, indicating to some extent that *M. nana* may be a species with a longer origin and evolutionary history than *M. rubra* in the genus *Myrica* Linn. Research has suggested that *M. nana* originated around at least 7 Ma which is more than 5.28 Ma for *M. rubra* (Liu et al., 2016), consistent with our results. Of course, other reasons such as demographic history and distribution range can also cause higher or lower genetic diversity. The phylogeographic results for *M. rubra* suggest that it underwent an obvious and extensive expansion and that southwest China is the refuge and origin center (Liu et al., 2016). Analysis of SDMs for *M. nana* based on ENM suggested that during the last glacial maximum (LGM), the distribution range of suitable area was contracted into two areas, consistent with the results of mismatch analysis which showed no expansion in the whole area during the LGM and the geographical distribution pattern of haplotype in our study. In addition, the findings of this study clearly show that there is an east–west divergence in *M. nana* populations, and there are unique or major chlorotypes occurring on either side of the Wumeng Mts, but there are some populations with two or more chlorotypes and higher genetic diversity. Thus each population or group is a microgene bank which has higher genetic diversity for *M. nana*.

### 4.2 Phylogeographical pattern and historical dynamics

Our study implies, based on cpDNA variation, the existence of an east–west chlorotype lineage split in *M. nana* populations, which correspond to geographic characters on each side of the Wumeng Mts. Chlorotype H2 had the highest distribution frequency in the east of Wumeng Mts, while the remaining chlorotypes were distributed on the west side of Wumeng Mts. In addition, the steps between chlorotypes H2 and H3 were up to 25, indicating that the east–west haplotype lineage split in *M. rubra* populations underwent a longer evolutionary history. Some nrDNA chlorotypes occurred across the Wumeng Mts, where the east–west genetic differences became smaller. This phylogeographic pattern may be due to pollen‐mediated high gene flow and secondary contact between east and west groups, which has been reported in other species on YGP and in the mountainous region of Southwest China (Gao et al., 2024; Ma et al., 2021). However, chlorotype h1 (13 individual plants) was mainly distributed in the east of the Wumeng Mts, and h3 (56 individual plants) exhibited a frequency of 68% and was mainly distributed in the west of the Wumeng Mts. This result supports the existence of an east–west chlorotype lineage split in *M. rubra* populations. The results of our AMOVA analysis showed that the significant variation for *M. rubra* populations was detected from within groups rather than from variation within populations. Chloroplast DNA is often used in phylogeography and evolution studies due to its characteristics of independent evolution, a relatively quick evolution of the non‐coding regions, and suitability for use in taxa (close relatives; Olmstead & Palmer, 1994; Sun et al., 2019; Wicke et al., 2011). Based on cpDNA variation, AMOVA analysis suggested that the genetic variation between the eastern populations (Group I) and western populations (Group II) was significantly different, and no chlorotypes were common to both lineages, which strongly supports the proposed split of Groups I and II in *M. rubra* populations. The east–west divergence in the genetic structure and the phylogeographical patterns further reveal the existence of natural geographical isolation boundaries caused by the Wumeng Mts on the YGP.

In terms of mismatch analysis, the actual curve for YGP populations exhibited multiple peaks, which is significantly different from the expected curve. The results showed that the *M. nana* populations experienced no obvious expansion in this region. Results of SDMs analysis for *M. nana* based on ENM suggested that during the LGM, the distribution range of suitable area was shrunk into at least two small regions, which is consistent with the results of mismatch analysis and the geographical distribution pattern of east–west chlorotype lineage split in whole distribution. It is suggested that geographic isolation and climate fluctuation may play an important role in influencing the phylogeographical pattern and historical dynamics of *M. nana*. Geographic isolation caused by the Wumeng Mts is obvious on the YGP, as it is located in the northeast of Yunnan Province and toward the west, northeast, and southwest of Guizhou Province. It shares a border between the Yunnan and Guizhou provinces, starting in the north and ending at the north border of Kunming. The YGP is divided into the Yunnan Plateau and the Guizhou Plateau by the Wumeng Mts. Furthermore, the YGP is one of the most typical limestone Karst landforms in the world, having the special isolated habitat of the Karst Islands (Xu, 2019; Zhao & Chen, 1999). There are significant differences in the geographical and environmental factors within the YGP. The range runs west to east, and the elevation of the terrain is uneven between 400 and 3500 m above sea level. The temperature, precipitation, and other environmental factors on the YGP also differ significantly: the temperature varies from high to low and decreases from west to east and from south to north; precipitation appears to be high in the east, west, and south and low in the center and north. The latest research reveals an obvious east–west divergence in genetic structure for *Amorphophallus yunnanensis*, *Juglans regia* subsp. *Sigillata* and *Urophysa rockii* and found that outliers are significantly related to environmental factors (e.g., altitudes) between east and west on the YGP and adjacent areas (Gao et al., 2024; Sun et al., 2019; Xie et al., 2017). The regional isolation and differences are significant, forming a unique topographic and ecological environment whose evolution has specific regional characteristics as well as global significance on the YGP (Tong et al., 1994; Xue et al., 2010). We also detected that *M. nana* experienced region population size expansion after 0.072 Ma taking BSP methods based on cpDNA datasets, which was happened during LGM. Thus these complex geographical and environmental factors and climate fluctuation in the past have had influences on the evolutionary history of vegetation on the YGP. The *M. nana* population has not experienced obvious population expansion overall, but we found similar or unique chlorotypes in most sub‐groups or areas indicating that small‐scale expansion events have occurred in small regions for *M. nana* populations. Thus, based on combined analysis of cpDNA and nrDNA sequences, mismatch analysis, BSP, and SDMs, our data reveal that the phylogeographical pattern, genetic diversity, genetic structure, and historical dynamics of *M. nana* populations can be attributed to the effects of geographic and environmental isolation and climate fluctuation on the YGP.

During the fourth glacial period, the whole world was affected by glacier movement and cold climate. The temperature during this period was 5–12°C lower than the current temperature and the glacier coverage area in China was 8.4 times more than it is now, which impacted all plants and animals considerably (Chen, 2011). The YGP was not greatly influenced during the fourth glacial ice coverage, but at the end of the last glacial climate change, vegetation on the YGP may have been affected. The geological development process, the uplift of the Qinghai–Tibet Plateau, and global climate change have led to a monsoon climate on the YGP, which is observed in east Asia and India, with the climate transition zone into a specific area (Li et al., 2013). In refuges, species migrate and survive during extreme environmental events. Thus, regions where species overlapped over the course of history are likely to be potential refuges for plants (Carnaval & Moritz, 2008), and they have higher genetic variation and genetic diversity. The Hengduan Mountains, distributed on the southeast margin of Qinghai‐Tibet Plateau and an adjacent area of YGP, was a typical shelter during the fourth glacial period and a transition zone for species contraction and expansion, as well as the origin and site of evolution of many species (Chen et al., 2019; Hu et al., 2022; Xie et al., 2017; Zhang et al., 2010). The current study, based on cpDNA and nrDNA fragments, suggests that there is an east–west divergence in the genetic structure and phylogeographical pattern of *M. nana*, further reveals the existence of a natural geographical isolation boundary of the Wumeng Mts on the YGP, and shows how the eastern populations are further divided into four subgroups. Thus, during the fourth glacial period, *M. nana* experienced significant environmental changes with at least two isolated refuges, which were distributed toward the east and west of the Wumeng Mts. These were insulated and divided by the natural geographical isolation boundary of the Wumeng Mts on the YGP. The two isolated refuges led to an east–west independent distribution pattern of chlorotypes and genetic splits. In addition, the results of SDMs analysis for *M. nana* based on ENM showed that the distribution range of suitable areas contracted during the LGM and present. Mismatch analysis also suggested that the *M. nana* population had experienced recent population contraction in its entire distribution and that small‐scale expansion events have occurred in small regions. These results expounded that geographic and environmental isolation and past climate fluctuation affected the phylogeographical pattern and historical dynamics of *M. nana*, and clarified that there were at least two isolated refuges for *M. nana* during the fourth glacial period. Thus, it is suggested that the *M. nana* populations within the YGP came from separate shelters within each region during the Quaternary ice age. In summary, geographic and environmental isolation and climate fluctuation in the past shaped the phylogeographical pattern, genetic structure, and historical dynamics of *M. nana* and contributed to the formation of at least two isolated refuges during the fourth glacial period on the YGP.

### 4.3 Protection strategy for *M. nana*

*M. nana*, also known as Yunnan bayberry, is an important wild plant resource endemic to the YGP and was once considered a vulnerable species. The fruit of *M. nana* is edible, and the root can be used as medicine, giving it high economic value (Liu et al., 2003; Xiang, 2005). In addition, *M. nana* is a species with good environmental and ecological benefits, as it can improve soil permeability and water storage and improve soil maturation and fertility; it is also fire‐resistant (Liu et al., 2003). *M. nana* is distributed in the special habitats of Karst limestone areas and is thus limited and scarce. During field sampling and investigation, we also noted the impact of environmentally destructive activities due to recent human actions, such as logging, mining, and fires, on the distribution and population size of *M. nana*. The illegal mining and selling of wild *M. nana* species have also caused great destruction. In our studies, SDMs analysis based on ENM showed that the suitable area for *M. nana* has contracted and will affect its distribution in the future. All of these give us a full picture of the challenges for protecting *M. nana*. Hence, it is imperative to take effective measures to protect *M. nana* in the present.

What effective measures we should take to protect *M. nana*? The protection of plant species is generally facilitated by employing on‐site conservation, namely dividing them into different geographical units at the site, which are based on special distribution regions for management and protection. Another method is off‐site conservation, which transplants the protected plant to a protected base such as a nature preserve or botanical garden. This protection measure requires that transplantation and naturalization of the protected plant cover different geographical units that retain the various characteristics of the plant. Different regional plants are affected by different environments and other factors influence their adaption and variation. Therefore, in addition to customizing the protection measures of a plant, efforts should also consider the plant's specific characteristics and habitats (Liu et al., 2020). Those two specified measures are effective and accessible. The findings of this study clearly show that the genetic structure, phylogeographical pattern, and historical dynamics of *M. nana* populations demonstrate an east–west divergence on the YGP, strengthening the idea that there were at least two isolated refuges during the Quaternary period, distributed between the east and west of the Wumeng Mts. The western populations are further divided into four groups, namely Panzhihua (north of the Yunnan Plateau), Dali (west of the Yunnan Plateau), Kunming (center of the Yunnan Plateau), and Wenshan areas (south of the Wumeng Mts). Thus, *M. nana* can be divided into two large gene banks from the perspective of germplasm preservation and geographical units, namely the Guizhou and Yunnan plateaus, distributed in east and west of the YGP, respectively, by the natural geographical isolation boundary of the Wumeng Mts. The western populations, located in the Yunnan Plateau, are further divided into at least four microgene banks from the perspective of germplasm preservation and geographical units. Effective and accessible protective measures for *M. nana* should be implemented as on‐site conservation in the Guizhou and Yunnan plateaus. We can also construct seed resource reserves in situ for *M. nana* in these two plateaus to maintain the existing population size, avoid the loss of chlorotype diversity caused by environmental changes, and improve the adaptability of the populations. In addition, off‐site conservation measures can be taken to protect *M. nana*. However, it must be ensured that off‐site conservation strategies for *M. nana* should transplant and preserve multiple resources and the construction of a resource bank must spread across all of the above‐mentioned geographical units and gene banks, especially for populations with high chlorotype diversity and unique chlorotypes, such as populations A, P, S, K, and M. Therefore, combined on‐ and off‐site conversation techniques can be employed to protect this important plant resource that is endemic to the YGP. Furthermore, variations in plant height and fruit colors and morphologies for *M. nana* should also be considered to ensure effective protection. What is more, tissue culture technique can effectively solve the problems of plant regeneration, breeding, cultivation, and promote the rapid growth of plants, which can be taken to effectively increasing the number of individuals for *M. nana*. Last but not least, raise residents' awareness about the conservation of *M. nana* resources, it is also a key to protecting this endemic species. In brief, *M. nana*, an important species endemic to the YGP has suffered great destruction and fragmentation, and it is imperative to take effective measures to protect it.

## 5 CONCLUSIONS

The phylogeographical pattern and genetic structure of 19 populations of *M. nana*, an important species endemic to the YGP, was investigated across the entire geographical distribution range of the YGP using two segments of chloroplast genes combined with the ITS sequence. The results indicate that there is east–west divergence in the genetic makeup of *M. nana* and the existence of at least two glacial refuges during the Quaternary glacial period, distributed on the east and west sides of the Wumeng Mts on the YGP, which further supports the existence of a natural geographical isolation boundary caused by the Wumeng Mts that divide the YGP into the Guizhou and Yunnan plateaus. The western populations are further divided into at least four groups. Thus, *M. nana* can be divided into two large gene banks for the preservation of germplasm and geographical units, namely, the Guizhou and Yunnan plateaus. The western populations, located in the Yunnan Plateau, are further divided into at least four microgene banks for the preservation of germplasm and geographical units. Combined on‐ and off‐site conservation techniques can be employed to protect *M. nana*, which has suffered considerable destruction and fragmentation due to various man‐made and natural factors. Overall, this study clarifies that the phylogeographical pattern and the genetic structure of *M. nana* can be attributed to geographic and environmental isolation as well as climate fluctuation, and outlines strategies for protecting *M. nana* species.

## AUTHOR CONTRIBUTIONS

**Min Wu:** Data curation (lead); formal analysis (lead); writing – original draft (lead). **Yu Cheng:** Data curation (lead); formal analysis (lead); investigation (lead); software (lead). **Chunxue Jiang:** Methodology (lead). **Mingsheng Zhang:** Supervision (supporting). **Tian Shi:** Investigation (supporting); methodology (supporting); software (supporting). **Cai Zhao:** Conceptualization (lead); data curation (lead); formal analysis (lead); funding acquisition (lead); investigation (lead); methodology (lead); project administration (lead); resources (lead); software (lead); supervision (lead); validation (lead); visualization (lead); writing – review and editing (lead).

## CONFLICT OF INTEREST STATEMENT

The authors declare no potential conflict of interest.

## Supporting information


Figure S1.
Figure S2.
Figure S3.
Figure S4.
Figure S5.
Figure S6.
Figure S7.
Figure S8.
Figure S9.

Table S1.
Table S2.
Table S3.

## Data Availability

ITS, *psbA*–*trnH*, and *trnD*–*psbM* sequences were deposited in the GenBank database under accession numbers QL468965–QL468997. Information on samples and genotype data can be inferred from a material table provided in our paper.
