# Supplementary material for: Phylogeography of Morella nana: The Wumeng Mountains as a natural geographical isolation boundary on the Yunnan‐Guizhou Plateau
Source: Ecol Evol. 2024 Jul 9;14(7):e11566. doi: 10.1002/ece3.11566 (PMC11232048; doi:10.1002/ece3.11566)
Supplement: Supplementary file 1 — Figure S1. Figure S2. Figure S3. Figure S4. Figure S5. Figure S6. Figure S7. Figure S8. Figure S9. [file ECE3-14-e11566-s001.docx]

**Supplementary Information**

**
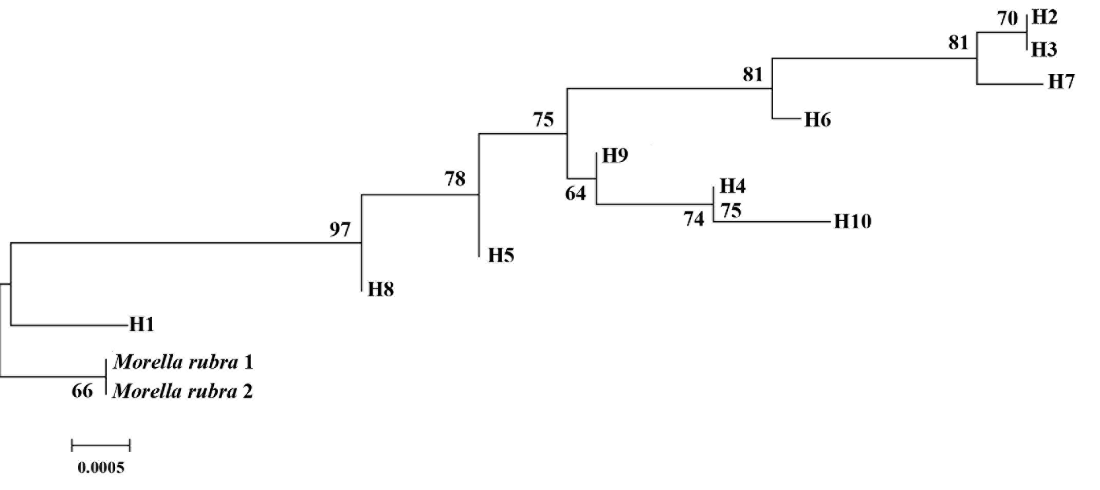
**

**Figure 1** Phylogenic relationships of 10 cpDNA chlorotypes in *M. Nana* and showed the actual branch lengths based on NJ. The bootstrap values is indicated above the tree branches. *M. rubra* is included as the outgroup. The bootstrap values less than 60％ is displayed.

**
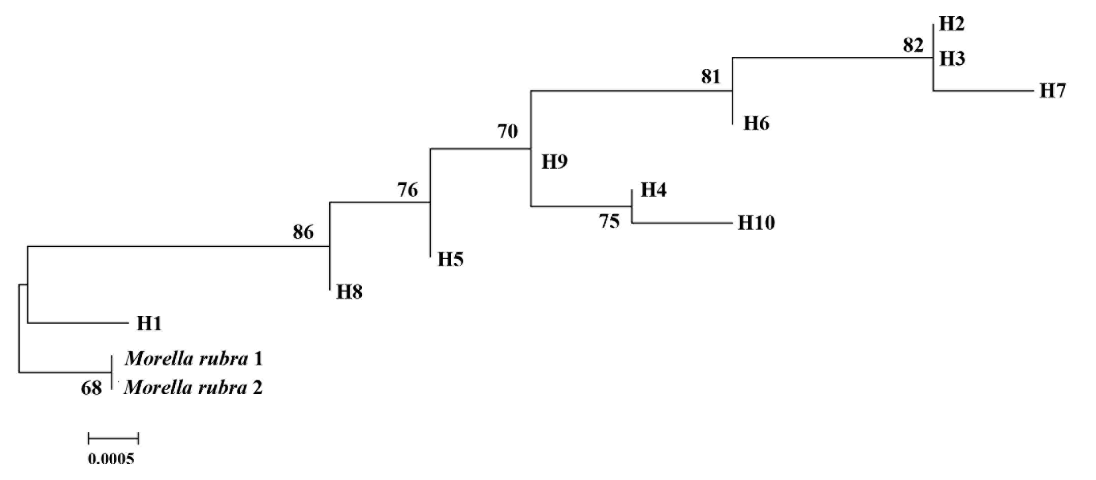
**

**Figure 2** Phylogenic relationships of 10 cpDNA chlorotypes in *M. Nana* and showed the actual branch lengths based on ML. The bootstrap values is indicated above the tree branches. *M. rubra* is included as the outgroup. The bootstrap values less than 60％ is displayed.

**
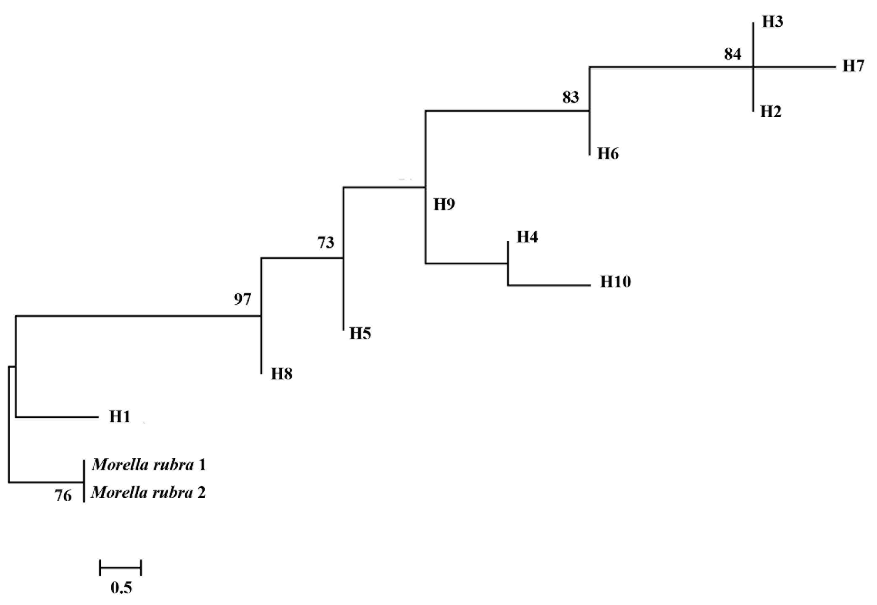
**

**Figure 3** Phylogenic relationships of 10 cpDNA chlorotypes in *M. Nana* and showed the actual branch lengths based on MP. The Bayesian posterior probabilities is indicated above the tree branches. *M. rubra* is included as the outgroup. The Bayesian posterior probabilities less than 60％ is displayed.

**
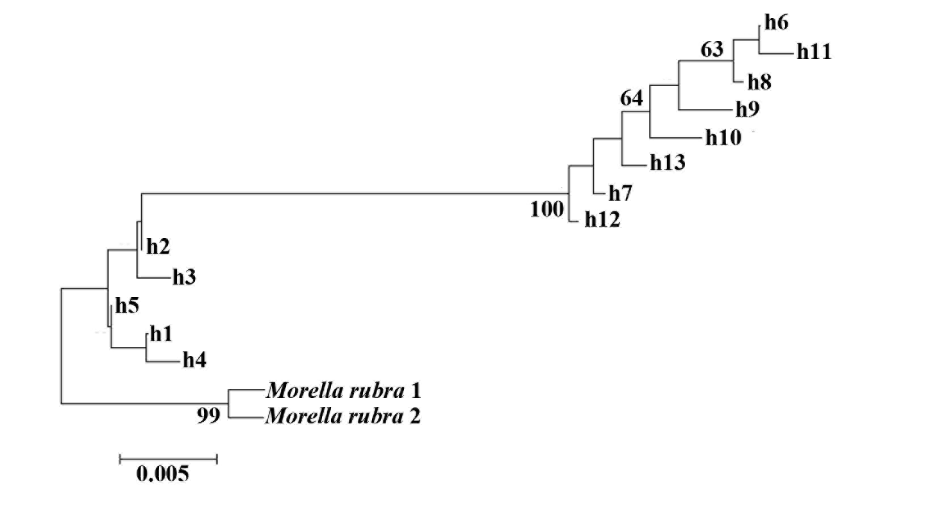
**

**Figure 4** Phylogenic relationships of 13 nrDNA (ITS) haplotypes in *M. Nana* and showed the actual branch lengths based on NJ. The bootstrap values is indicated above the tree branches. *M. rubra* is included as the outgroup. The bootstrap values less than 60％ is displayed.


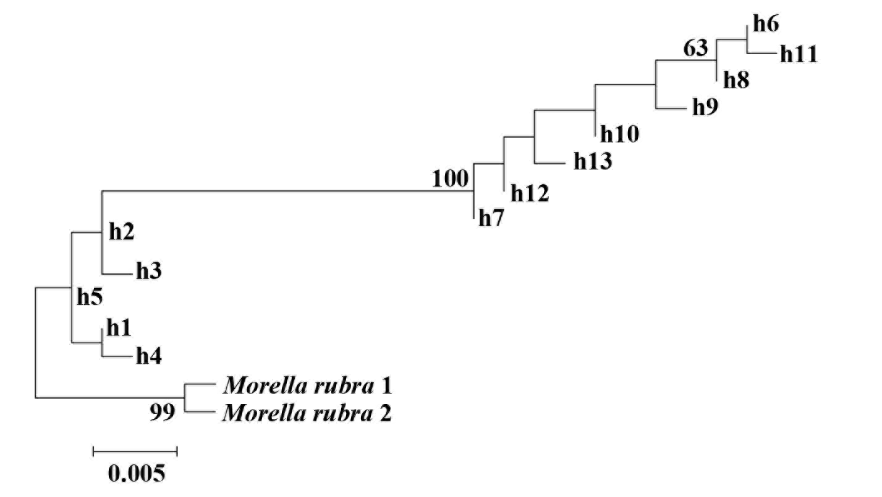


**Figure 5** Phylogenic relationships of 13 nrDNA (ITS) haplotypes in *M. Nana* and showed the actual branch lengths based on ML. The bootstrap values is indicated above the tree branches. *M. rubra* is included as the outgroup. The bootstrap values less than 60％was displayed.


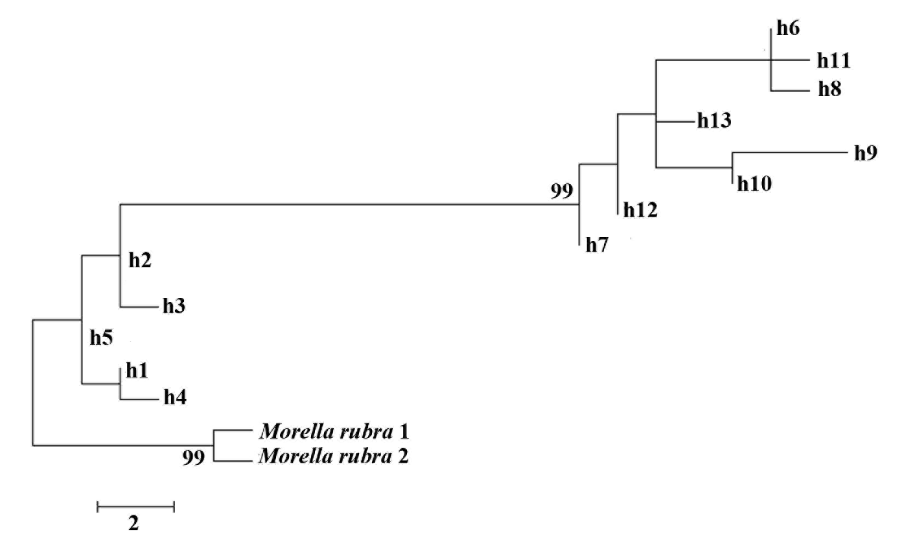


**Figure 6** Phylogenic relationships of 13 nrDNA (ITS) haplotypes in *M. Nana* and showed the actual branch lengths based on MP. The bootstrap values is indicated above the tree branches. *M. rubra* is included as the outgroup. The Bayesian posterior probabilities less than 60％ is displayed.

**

**

**Figure 7**  Phylogenic relationships of 10 cpDNA chlorotypes in *M. Nana.* The bootstrap values and Bayesian posterior probabilities are indicated above the tree branches in the order NJ/ML/MP. The bootstrap values and Bayesian posterior probabilities less than 60％was displayed by “*”. *M. rubra* is included as the outgroup.


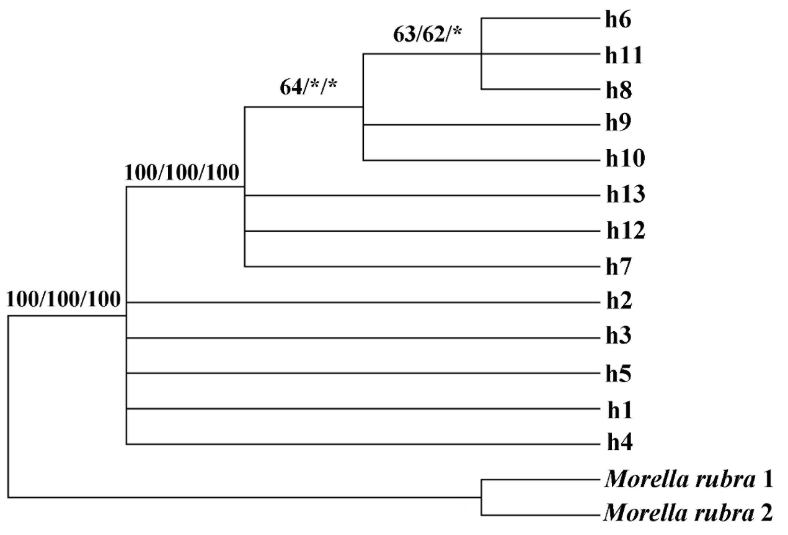


**Figure 8** Phylogenic relationships of 13 nrDNA (ITS) haplotypes in*M. Nana.* The bootstrap values and Bayesian posterior probabilities are indicated above the tree branches in the order NJ/ML/MP. The bootstrap values and Bayesian posterior probabilities less than 60％ was displayed by “*”. *M. rubra* is included as the outgroup.


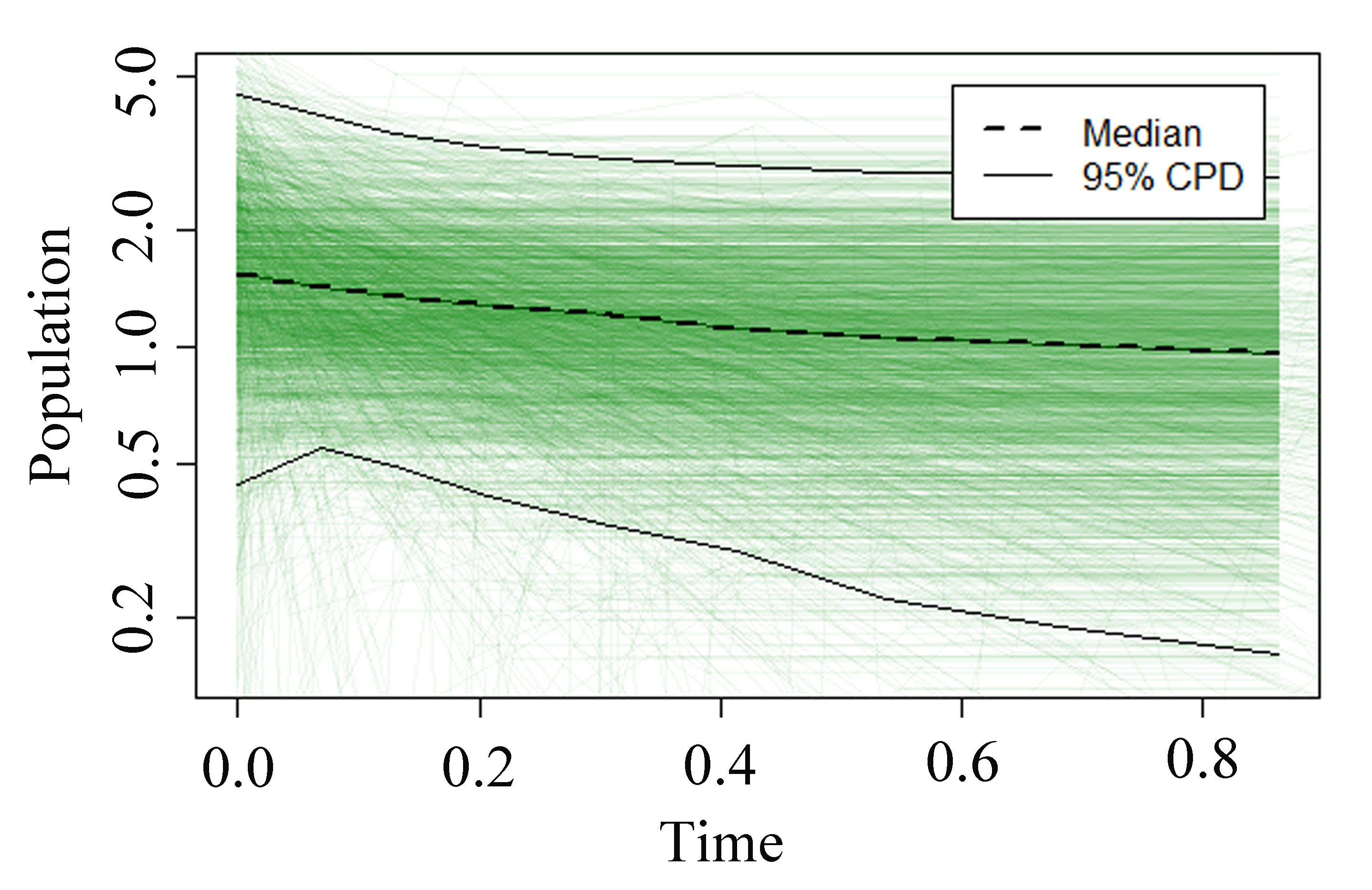


**Figure 9**  Bayesian skyline plot for *M. rubra* populations. The black dashed line indicates the median values of effective population size (*Ne*). The upper and lower black lines represent the boundaries of the highest 95% posterior density (HPD) interval of Ne.
