# Supplementary material for: Phylogeography of Morella nana: The Wumeng Mountains as a natural geographical isolation boundary on the Yunnan‐Guizhou Plateau
Source: Ecol Evol. 2024 Jul 9;14(7):e11566. doi: 10.1002/ece3.11566 (PMC11232048; doi:10.1002/ece3.11566)
Supplement: Supplementary file 2 — Table S1. Table S2. Table S3. [file ECE3-14-e11566-s002.docx]

**Supporting Information**

Table 1 Primer and protocols of PCR for *M. nana.*

| primer sequence | Primer (5′-3′) | PCR protocol |
| --- | --- | --- |
| ITS | F: GGAAGTAAAAGTCGTAACAAGG  R: TCCTCCTCCGCTTATTGATATGC | 94℃, 3min; 29×(94℃, 30s; 53.5℃, 30s; 72℃, 1min); 72℃, 5min |
| *psbA-trnH* | F: GTTATGCATGAACGTAATGCTC  R: AGTACCGATTGATAGGAGAA | 94℃, 3min; 31×(94℃, 30s; 58℃, 30s; 72℃, 1min); 72℃, 5min |
| *trnD-psbM* | F: CCACCTTTACACTTATTTAC  R: AGTACCGATTGATAGGAGAA | 94℃, 3min; 31×(94℃, 30s; 58℃ ,30s; 72℃, 1min); 72℃, 5min |

Table 2 Polymorphic site of each haplotype for *M. nana* in the cpDNA (*psbA-trnH*+*trnD-psbM*) sequences.

| haplotypes | Nucleotide position | | | | | | | | | | | | | | |
| --- | --- | --- | --- | --- | --- | --- | --- | --- | --- | --- | --- | --- | --- | --- | --- |
|  | 48 | 92 | 189 | 227 | 283 | 334 | 340 | 341 | 429 | 630 | 702 | 904 | 927 | 935 | 961 |
| H1 | T | G | A | * | A | A | G | A | T | C | C | T | T | T | - |
| H2 | T | A | G | - | C | C | T | C | - | T | T | T | G | C | + |
| H3 | T | A | G | - | C | C | T | C | - | T | T | T | G | C | - |
| H4 | G | A | G | - | A | C | G | A | - | T | C | T | G | C | - |
| H5 | T | A | G | - | A | C | G | A | - | T | C | T | T | C | - |
| H6 | T | A | G | - | A | C | T | C | - | T | C | T | G | C | - |
| H7 | T | A | G | - | C | C | T | C | - | T | T | T | T | C | - |
| H8 | T | A | G | - | A | A | G | A | - | T | C | T | T | C | - |
| H9 | T | A | G | - | A | C | G | A | - | T | C | T | G | C | - |
| H10 | G | A | G | - | A | C | G | A | - | T | C | C | G | C | - |

Notes:“*”equate to“TATTAA”; “+”equate to“ATGTAATTTATACTACATGTAGTA”; “**-**”equate to the gap

Table 3 Polymorphic site of each haplotype for *M. nana* in the nrDNA sequences.

| haplotypes | Nucleotide position | | | | | | | | | | | | | | | | | | | | | | |
| --- | --- | --- | --- | --- | --- | --- | --- | --- | --- | --- | --- | --- | --- | --- | --- | --- | --- | --- | --- | --- | --- | --- | --- |
|  | **1** | **9** | **10** | **13** | **14** | **76** | **85** | **88** | **99** | **170** | **227** | **283** | **312** | **319** | **322** | **359** | **392** | **414** | **424** | **450** | **484** | **503** | **521** |
| H1 | G | A | C | A | G | C | C | C | C | C | C | C | C | C | C | C | T | C | C | C | T | C | A |
| H2 | G | A | C | A | G | C | C | C | C | C | C | C | C | C | C | C | C | C | C | C | C | C | A |
| H3 | G | A | C | A | G | C | C | C | C | C | C | C | C | C | C | C | C | C | C | C | C | C | G |
| H4 | G | A | C | A | G | C | C | C | C | C | C | C | C | C | C | C | T | C | C | C | T | C | G |
| H5 | G | A | C | A | G | C | C | C | C | C | C | C | C | C | C | C | C | C | C | C | T | C | A |
| H6 | A | G | A | C | A | T | T | T | T | T | T | T | C | T | T | T | C | T | T | T | C | C | A |
| H7 | A | G | A | C | A | C | T | C | T | T | T | T | C | T | C | C | C | T | T | C | C | C | A |
| H8 | A | G | A | C | A | T | T | T | T | T | T | T | C | T | T | T | C | T | T | T | C | T | A |
| H9 | A | G | C | C | A | C | T | T | T | T | T | T | G | T | T | T | C | T | T | T | C | T | A |
| H10 | G | G | A | C | A | C | T | C | T | T | T | T | G | T | T | C | C | T | T | T | C | T | A |
| H11 | G | G | A | C | A | T | T | T | T | T | T | T | C | T | T | T | C | T | T | C | C | C | A |
| H12 | A | G | A | C | A | C | T | C | T | T | T | T | C | T | C | C | C | T | T | T | C | C | A |
| H13 | A | G | C | C | A | C | T | C | T | T | T | T | C | T | T | C | C | T | T | T | C | C | A |
